# Exploring barriers and facilitators to shared decision-making for older adults in East Asian health systems: a scoping review

**DOI:** 10.1093/geroni/igag080

**Published:** 2026-07-10

**Authors:** Seung Eun Lee, Misun Hwang, Yun Jiang

**Affiliations:** Mo-Im Kim Nursing Research Institute, College of Nursing, Yonsei University, Seoul, South Korea; School of Nursing, University of Michigan, Ann Arbor, Michigan, United States; School of Nursing, University of Michigan, Ann Arbor, Michigan, United States

**Keywords:** Culture, Family, Person-centered care, Health Care Systems & Management

## Abstract

**Background and Objectives:**

Shared decision-making (SDM) is a core component of patient-centered care, yet evidence on SDM among older adults is largely derived from Western healthcare systems. Given sociocultural differences in East Asian contexts, particularly regarding family involvement, the transferability of Western SDM models remains uncertain. This study aimed to identify barriers and facilitators to SDM for older adults in East Asian healthcare systems across stakeholder groups.

**Methods:**

A scoping review was conducted following the Arksey and O’Malley framework and PRISMA-ScR guidelines. Six databases were searched from inception to October 2025 for studies focused on older adults (≥65 years) in East Asian settings. Findings were synthesized descriptively and organized across 4 stakeholder groups: patients, caregivers, clinicians, and health systems.

**Results:**

Twenty studies published between 2011 and 2025 were included. SDM was most examined in end-of-life care, life-sustaining treatment decisions, and advance care planning. Patient-related barriers included limited information and literacy, poorer health and financial burden, psychological distress, and constrained autonomy. Caregiver-related factors reflected family-dominated decision-making dynamics that could both hinder and support SDM. Barriers regarding clinicians included clinician-centered practices, limited interaction, and delayed discussions, while those in health systems included insufficient decision-support infrastructure and resource constraints.

**Discussion and Implications:**

SDM for older adults in East Asia is shaped by interacting influences involving patients, caregivers, clinicians, and health systems, with family involvement playing a central contextual role. Culturally responsive SDM approaches and research beyond end-of-life contexts are needed to support meaningful participation in clinical decision-making across care settings.

Innovation and Translational Significance: Despite its crucial role in patient-centered care, evidence on shared decision-making (SDM) among older adults is limited to Western healthcare settings. This review synthesizes evidence on barriers and facilitators to SDM among older adults across key stakeholder groups, including patients, caregivers, clinicians, and health systems, within East Asian healthcare contexts. Findings underscore the roles of family involvement, clinician authority, and structural constraints, such as limited consultation time, high patient volume, and cultural norms. Future research should extend SDM beyond end-of-life care and examine culturally responsive strategies to support meaningful participation in clinical decisionmaking across East Asian contexts.

## Background and objectives

Shared decision-making (SDM) is globally recognized as a core component of patient-centered care. Commonly defined as a collaborative process in which patients and healthcare professionals work together to reach informed decisions about diagnosis, treatment, and ongoing management, SDM involves key steps such as presenting options, discussing potential benefits and harms, and eliciting and integrating patient values and preferences.^1^^,^^2^ Successful SDM has been consistently associated with positive patient outcomes, including greater satisfaction, improved trust in clinicians, and better symptom management.^3^^,^^4^ Reflecting this evidence, there has been a significant movement to integrate SDM into routine clinical practice and health policy. Countries such as the United Kingdom, through the National Institute for Health and Care Excellence (NICE) guidelines,^5^ and the United States, through the Agency for Healthcare Research and Quality (AHRQ)’s SHARE approach,^6^ have developed national guidelines, training curricula, and patient decision aids to institutionalize SDM within health system reforms that emphasize patient autonomy.

Despite the global recognition of SDM, its conceptualization and implementation in East Asian health contexts remain comparatively underexplored. Healthcare systems are embedded within distinct sociocultural and clinical contexts, which shape how SDM is understood and practiced; as a result, Western-derived models cannot be assumed to translate seamlessly to East Asian settings.^7^ In this region, contextual differences often manifest as structural and cultural constraints within the clinical environment, including high patient volumes, brief consultation times, and hierarchical norms shaping patient–clinician communication, often characterized by clinician authority, patient deference, and more directive communication styles.^8^^,^^9^ These factors collectively limit the time, flexibility, and relational space required to engage in comprehensive SDM processes.^10^ Although interest in patient-centered care and SDM is growing in East Asia, empirical evidence on the specific barriers and facilitators of SDM in this region remains limited.^10^

To move beyond generic application and develop context-sensitive implementation strategies, there is a critical need to identify and map the barriers and facilitators that influence SDM for the older adult population in East Asian settings. Older adults represent a particularly important population in East Asian healthcare systems, where rapid population aging and increased longevity have led to a growing demand for complex healthcare decision-making.^11^ They face compounded challenges, including the accumulation of multiple chronic conditions, poorer overall health status, and age-related cognitive impairments, all of which may compromise the capacity to engage in and participate fully in decision-making processes.^12^ In addition, broader social and demographic changes, including shifts in family structures and caregiving patterns, may further influence the context in which these decisions occur.^13^ Tensions between cultural duties such as filial piety and individual patient autonomy are often especially pronounced, particularly in family-mediated or surrogate decision-making around serious illness and end-of-life care.^9^^,^^14^ These intersecting clinical, cultural, and relational factors highlight the importance of examining SDM not only at the level of individual communication skills but also within broader family and system-level dynamics.

East Asian contexts remain largely unrepresented in the existing literature on barriers and facilitators to SDM among older adults. A previous systematic review examining SDM among older adults with multiple chronic conditions identified several barriers and facilitators. Reported barriers included poor health, cognitive or physical impairments, and organizational constraints such as time pressure, whereas facilitators included explicitly inviting patients to participate in decision-making, emphasizing individual values and preferences, and supporting patient autonomy in clinical decisions.^12^ However, the studies included in that review were conducted primarily in Western countries, including the United States, European countries, and Australia, which may limit the applicability of these findings to East Asian contexts.^12^

In many East Asian societies, medical decision-making is shaped by collectivistic family norms and the moral weight of filial obligations, such that the family unit rather than the individual patient is often regarded as the primary locus of decision-making authority.^15^^,^^16^ These Confucian-derived norms of family harmony and filial piety have been shown to influence prognosis disclosure, advance care planning, and decisions about life-sustaining treatments, as well as the extent to which patient autonomy is foregrounded in clinical encounters.^15^^,^^17^ While some of these sociocultural features may also be observed in other parts of Asia, they have been particularly well described in East Asian contexts.^15^^,^^18^ These sociocultural characteristics may shape how SDM is practiced among older adults in East Asia, highlighting the need for a focused examination of SDM barriers and facilitators in this region.

The present scoping review aims to identify and categorize key barriers and facilitators to SDM within East Asian contexts. Specifically, this review seeks to answer the following research question: *What barriers and facilitators to SDM for older adults have been reported in East Asian health systems, and how can they be categorized across key stakeholder domains (patients, caregivers, clinicians, and the health system)?* By synthesizing the available evidence across these 4 critical domains, this review will provide a foundational evidence base for the development of culturally attuned models and intervention strategies tailored to aging populations in East Asia.

## Research design and methods

This scoping review was conducted following the methodological framework proposed by Arksey and O’Malley^19^ and further refined by the Joanna Briggs Institute.^20^ This review is reported in accordance with the Preferred Reporting Items for Systematic Reviews and Meta-Analyses extension for Scoping Reviews (PRISMA-ScR).^21^ The protocol for this review was preregistered on the Open Science Framework (OSF) (https://osf.io/tx29b/overview)

To guide study selection, eligibility criteria were defined using the Population–Concept–Context framework recommended for scoping reviews. The population of interest was adults aged 65 years and older receiving healthcare in East Asian countries and regions. Although various international definitions of older adults exist, this review adopted 65 years as the threshold, consistent with demographic reporting practices in East Asia and widely used international aging indicators.^11^ Where explicitly reported, studies were included if they explicitly targeted participants aged 65 years or older. Studies were also considered eligible when the reported mean or median age of the sample was 65 years or older, following the eligibility criteria used in a previous systematic review of SDM in older adults with multiple chronic conditions.^12^ The concept of interest was SDM, specifically reported barriers and facilitators to SDM. The context included any healthcare setting within East Asian health systems. For the purpose of this review, East Asia was defined based on commonly used geographic classifications (eg, United Nations Statistics Division) and included the following countries and regions: China, Japan, Korea, Taiwan, Hong Kong, Macau, and Mongolia.^22^ Detailed search strategies are provided in the Supplementary Methods.

A comprehensive literature search was conducted from database inception to October 2025 in CINAHL, Embase, Global Health, PubMed, Scopus, and Web of Science. The search strategy combined controlled vocabulary and keywords related to older adults, SDM, barriers and facilitators, and East Asian healthcare systems. The strategy was developed with support from a health sciences librarian and iteratively refined to ensure comprehensive retrieval. The search was limited to English-language publications. Studies were excluded if their primary aim was to evaluate the effectiveness of a specific intervention without addressing barriers or facilitators of SDM. Non-original research articles, including conference abstracts, study protocols, reviews, commentaries, and instrument development studies, were also excluded. Search results were imported into Covidence, where duplicates were removed prior to screening. Two reviewers (SL and MH) independently screened titles and abstracts, followed by full-text reviews to determine eligibility. Discrepancies were resolved through discussion.

Data were extracted using a piloted data-charting form. Extracted information included bibliographic details, study aim, design, setting, participant characteristics, decision-making focus, conceptualization of SDM, and reported barriers and facilitators related to SDM. The data were synthesized descriptively using a deductive approach, with barriers and facilitators categorized into stakeholder domains, including patients, caregivers, clinicians, and health system factors. This categorization reflects a multilevel perspective on healthcare decision-making, consistent with prior research examining barriers and facilitators to SDM across multiple levels.^12^ Within each domain, findings were synthesized to capture the range of reported barriers and facilitators across studies.

## Results

### Study selection

**Figure 1:**
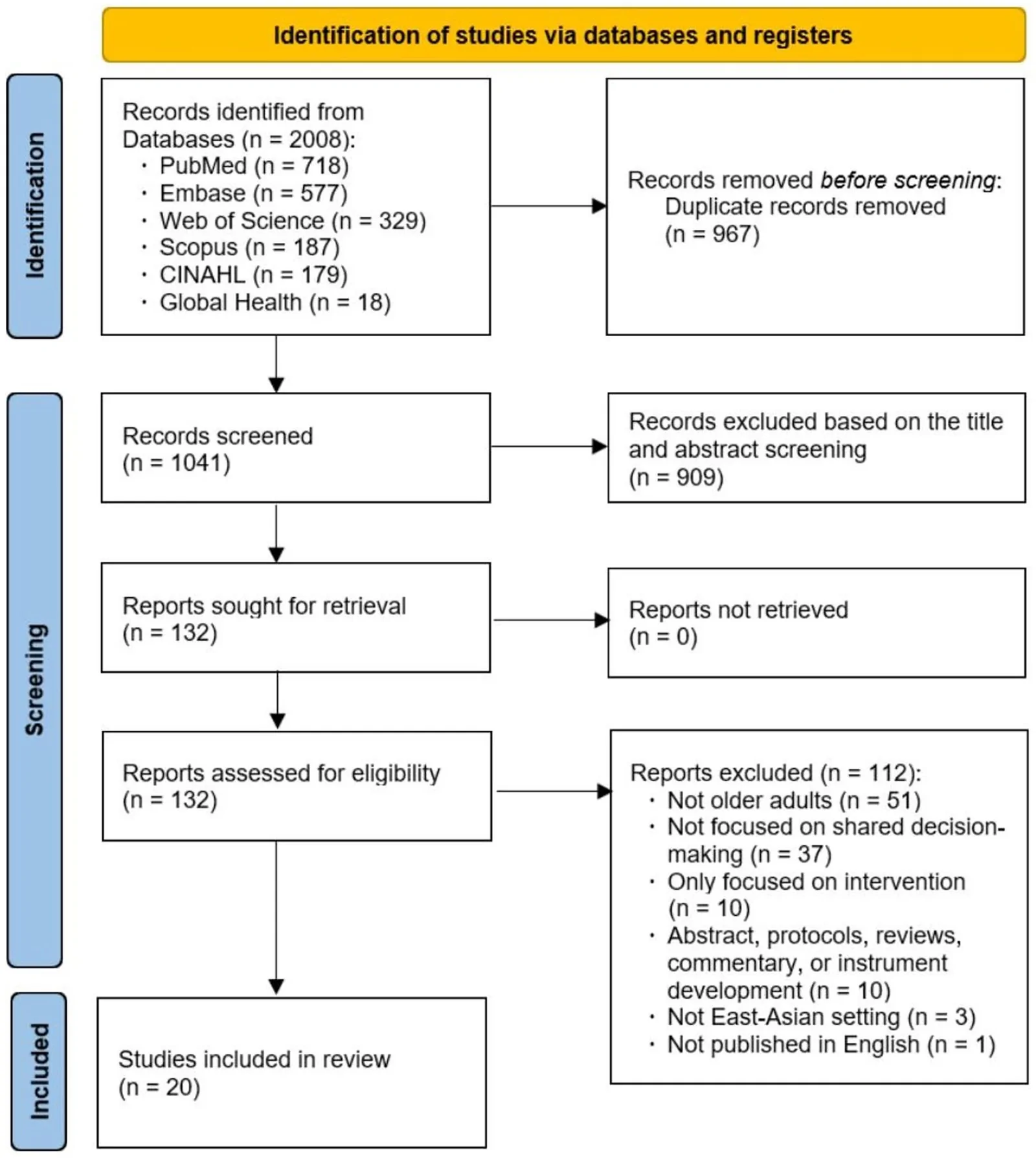
PRISMA flow diagram.

The literature search across 6 databases identified 2008 records, of which 967 duplicates were removed. After title and abstract screening, 132 articles remained for full-text reviews. As a result, 20 studies were included in the final review and analysis. The study selection process is summarized in the PRISMA flow diagram (Figure 1).

### Study characteristics

As shown in Table 1, the 20 included studies were published between 2011 and 2025. Although the eligibility criteria allowed studies from East Asian countries and regions, the included studies were conducted in China (*n = *8),^23–30^ Taiwan (*n = *5),^31–35^ Japan (*n = *4),^36–39^ South Korea (*n = *2),^40^^,^^41^ and Hong Kong (*n = *1).^42^ The included studies employed diverse designs, including qualitative studies (*n = *8),^23^^,^^25–27^^,^^29^^,^^33^^,^^34^^,^^40^ quantitative observational studies (*n = *11),^24^^,^^30–32^^,^^35–39^^,^^41^^,^^42^ and 1 mixed-method study.^28^ Sample sizes ranged from 12 to 1600 participants, and the reported mean or median age of participants ranged from 65 to 86.9 years. Regarding the primary focus of decision-making, the studies addressed SDM in treatment decisions (*n = *9),^23^^,^^25–27^^,^^33^^,^^37^^,^^38^^,^^40^^,^^41^ advance care planning (ACP) (*n = *4)^28^^,^^34–36^ or advance directives (AD) (*n = *2),^32^^,^^42^ end-of-life (EOL) care (*n = *4),^24^^,^^29^^,^^31^^,^^39^ and life-sustaining treatment (LST) decision (*n = *1).^30^ Further details on study characteristics are provided in Supplementary Table 1.

**Table 1:** Characteristics of included studies (*N = *20).

References	Country or region (setting)	Study design	Study population (sample size)	Mean or median age	Primary focus of decision-making
Cao et al.^23^	China (tertiary hospital)	Qualitative	Patients with prostate cancer (*n = *34) Medical and nursing staff (*n = *16)	65.3 (mean)	Treatment decision (prostate cancer surgery)
Chen et al.^31^	Taiwan (tertiary public hospital)	Quantitative (secondary analysis)	Patients with cancer who died in a hospital (*n = *1338)	66.8 (mean)	End-of-life care (terminal cancer care)
Chong et al.^40^	South Korea (university hospital)	Qualitative	Patients underwent kidney transplantation (*n = *12)	67.4 (mean)	Treatment decision (kidney transplantation)
Chu et al.^32^	Taiwan (public hospital)	Quantitative (secondary analysis)	Patients with chronic life-limiting illness or functional impairment (*n = *1411)	78.2 (mean)	Advance directive
Chu et al.^42^	Hong Kong (nursing homes)	Quantitative	Older adults residing in nursing homes (*n = *1600)	82.4 (mean)	Advance directive
Chuang et al.^33^	Taiwan (regional hospital)	Qualitative	Patients with degenerative joint disease (*n = *21)	69.9 (mean)	Treatment decision
Fang et al.^24^	China (nursing home)	Quantitative	Older adults with disability, dementia, or those over 80 years (*n = *571)	86.9 (mean)	End-of-life care
Guo et al.^25^	China (tertiary hospital)	Qualitative	Patients with stroke (*n = *31)	Not reported^a^	Treatment decision (rehabilitation)
Hsu et al.^34^	Taiwan (community center)	Qualitative	Patients with chronic disease (*n = *13) Family caregivers (*n = *12) Community nurses (*n = *11)	71.7 (mean)	Advance care planning
Kanakubo et al.^36^	Japan (medical institutions)	Quantitative	Patients undergoing hemodialysis (*n = *453)	69 (mean)	Advance care planning
Komatsu et al.^37^	Japan (communities)	Quantitative (secondary analysis)	Older adults covered by long-term care insurance (*n = *643) Primary caregivers (*n = *643)	Not reported^b^	Treatment decision (long-term care)
Lin et al.^35^	Taiwan (communities)	Quantitative	Patients receiving home-based medical care (*n = *408)	80.4 (mean)	Advance care planning
Liu et al.^26^	China (tertiary hospital)	Qualitative	Patients with multiple comorbidities (*n = *12) Caregivers (*n = *9) Healthcare professionals (*n = *7)	65 (mean)	Treatment decision
Shibagaki et al.^38^	Japan (medical institutions)	Quantitative	Patients with chronic kidney disease (*n = *475)	67.4 (mean)	Treatment decision (renal replacement therapy)
Shin et al.^41^	South Korea (hospitals)	Quantitative	Patients with cancer (*n = *358) Family caregivers (*n = *358)	71.1 (mean)	Treatment decision
Shinada et al.^39^	Japan (tertiary hospitals)	Quantitative	Caregivers who experienced the loss of older patients with cardiovascular disease (*n = *266)	82 (median)	End-of-life care
Wang et al.^27^	China (tertiary hospitals)	Qualitative	Patients who have undergone coronary artery bypass grafting (*n = *19)	72.2 (mean)	Treatment decision (cardiac rehabilitation)
Yang et al.^28^	China (communities)	Mixed-method	Patients with chronic diseases -survey (*n = *471) -interview (*n = *14)	Not reported^a^	Advance care planning
Ye et al.^29^	China (nursing homes)	Qualitative	Patients with chronic diseases or cancer (*n = *20)	77.8 (mean)	End-of-life care (hospice and palliative care)
Zhu et al.^30^	China (communities)	Quantitative	Patients with chronic conditions (*n = *150) Family caregivers (*n = *150)	71 (mean)	Life-sustaining treatment decision

a More than 50% of participants were aged ≥70 years.

b All participants were aged ≥65 years.

### Conceptualization of shared decision-making

Table 2 summarizes the stakeholder groups considered in the SDM process (patients, caregivers, clinicians, and the health system) and how SDM was described across the included studies. The studies were further categorized according to their primary decision-making focus (treatment decisions, ACP/AD, and EOL/LST decisions). About half of the studies (*n = *8) considered all 4 stakeholder groups in the SDM process,^23^^,^^25–27^^,^^29^^,^^31^^,^^34^^,^^40^ while 3 studies focused only on patient-related factors.^24^^,^^35^^,^^42^

**Table 2:** Conceptualization of shared decision-making (*N = *20).

References	Stakeholder groups considered	Description of SDM
**Studies addressing treatment decisions (*n* = 9)**		
Cao et al.^23^	Patients, caregivers, clinicians, system	A deliberative process involving access to information, shared treatment goals, and understanding of treatment consequences between patients and clinicians
Chong et al.^40^	Patients, caregivers, clinicians, system	An approach involving active support for patients in making decisions that reflect their autonomy and values, thereby maximizing their right to self-determination.
Chuang et al.^33^	Patients, caregivers, clinicians	A patient-centered approach involves patient-clinician communication to clarify evidence-based practice and consider patients’ preferences.
Guo et al.^25^	Patients, caregivers, clinicians, system	A collaborative approach involving patients and clinicians that integrates the best evidence, patient preferences, and values to help patients make informed health decisions.
Komatsu et al.^37^	Patients, caregivers	A process involving patients’ participation and engagement in daily care decisions and choices in receiving care.
Liu et al.^26^	Patients, caregivers, clinicians, system	A process involving the participation of patients and healthcare professionals in decision-making, considering patients’ values, preferences, and actual circumstances.
Shibagaki et al.^38^	Patients, system	A collaborative approach involving clinicians and patients in choosing treatment options, considering patients’ circumstances, values, and goals.
Shin et al.^41^	Patients, caregivers	A process involving patients’ ability to understand and use medical information to make treatment decisions aligned with their values and preferences.
Wang et al.^27^	Patients, caregivers, clinicians, system	A collaborative approach involving patients and healthcare professionals in decision-making, considering patients’ background, goals, values, and preferences to achieve desired treatment outcomes.
**Studies addressing ACP/AD (*n* = 6)**		
Chu et al.^32^	Patients, system	A process involving AD discussions as part of ACP for patients who may lose decision-making capacity.
Chu et al.^42^	Patients	A process involving AD discussions about the type of health care patients would want if they are no longer competent.
Hsu et al.^34^	Patients, caregivers, clinicians, system	A process involving ACP to understand patients’ preferences, improve symptom management, and provide patient-centered care.
Kanakubo et al.^36^	Patients, clinicians	A process involving ACP discussions and documentation of patients’ goals and preferences for future medical care.
Lin et al.^35^	Patients	A process involving ACP discussions to clarify patients’ medical decisions and respect their voices in treatment and care preferences, thereby facilitating patient-centered care.
Yang et al.^28^	Patients, caregivers, system	A process involving ACP that requires patients to express their preferences for treatment and care in case their condition deteriorates, based on their life experiences and values.
**Studies addressing EOL care/LST (*n* = 5)**		
Chen et al.^31^	Patients, caregivers, clinicians, system	A process involving DNR orders, assuming prior disclosure of terminal status and end-of-life options, and using a signed DNR letter of intent as an indicator of patient autonomy.
Fang et al.^24^	Patients	A process involving EOL care that is influenced by both clinical and psychosocial factors, as well as individuals’ perceived control over treatment outcomes.
Shinada et al.^39^	Patients, caregivers	Not reported
Ye et al.^29^	Patients, caregivers, clinicians, system	Not reported
Zhu et al.^30^	Patients, caregivers	A process involving LST discussions as part of ACP, enabling patients to discuss their goals and preferences for future care with their families and clinicians.

Abbreviations: ACP, advance care planning; AD, advance directives; DNR, do-not-resuscitate; EOL, end-of-life; LST, life-sustaining treatment; SDM, shared decision-making.

Among studies focusing on treatment decisions,^23^^,^^25–27^^,^^33^^,^^37^^,^^38^^,^^40^^,^^41^ SDM was commonly described as a collaborative, patient-centered process involving information exchange, deliberation between patients and clinicians, and the alignment of treatment decisions with patients’ goals, preferences, and values. In studies examining ACP or AD,^28^^,^^32^^,^^34–36^^,^^42^ decision-making was conceptualized primarily as preparation for future medical decisions, active engagement in planning discussions, and the documentation or completion of AD to ensure care that aligns with patients’ values and preferences. Studies addressing EOL or LST decisions^24^^,^^29–31^^,^^39^ conceptualized decision-making through constructs such as patient autonomy, involvement of family caregivers, and deliberation regarding treatment options within EOL contexts.

### Barriers and facilitators to shared decision-making

Table 3 presents a simplified summary of barriers and facilitators to SDM across key stakeholder groups (patients, caregivers, clinicians, and the health system) in the included studies. Detailed items are provided in Supplementary Table 2.

**Table 3:** Barriers and facilitators to shared decision-making at multiple stakeholder groups.

Stakeholder groups	Barriers	Facilitators
Patient	Limited information, health literacy, and understanding Personal health, situational, and resource-related challenges Psychological and emotional distress Constrained autonomy and interactions with clinicians Avoidance or reluctance toward EOL discussion	Greater individual capacity and self-efficacy Personal health, situational, and resource-related support Information preferences, access, and learning opportunities Supportive social discussion Prior experience-based awareness Personal values favoring dignity in care
Caregiver	Family-led decision-making dynamics Avoidance and limited support Lack of caregiver preparedness, knowledge, and health	Shared involvement and respect for patients Detailed communication and understanding of the clinical situation
Clinician	Lack of communication skills and discomfort with uncertainty Clinician-centered decision-making practices Limited interaction and emotional support Delayed timing of EOL decisions	Patient-centered communication practices Continuity of care
Health system	Lack of decision-support infrastructure Resource and structural constraints Fragmentation and lack of care integration Information barriers and complexity Care-setting constraints	Palliative care and multidisciplinary support Community integration High facility capacity or volume Quality and stability of the care environment

Abbreviation: EOL, end-of-life.

#### Patient-related factors

Among patient-related factors, barriers to SDM were commonly related to patients’ limited information, health literacy, and understanding of their disease and treatment options,^23^^,^^25–27^^,^^29^^,^^31^^,^^40^ along with other personal health, situational, and resource-related challenges, such as complex health and care needs or financial burden.^26^^,^^29^^,^^31^^,^^37–40^^,^^42^ Psychological and emotional distress^23^^,^^25^^,^^27^^,^^28^^,^^40^ and constrained autonomy and interactions with clinicians^25–27^^,^^33^^,^^40^ were also highlighted as limiting patients’ ability to actively engage in decision-making. In a few studies, avoidance or reluctance toward EOL discussion due to religious beliefs or attitudes toward death was also reported.^29^^,^^34^^,^^40^

Patient-related facilitators were frequently associated with greater individual capacity and self-efficacy, such as high educational level or self-care ability,^24^^,^^26^^,^^28^^,^^35^^,^^41^^,^^42^ as well as several personal health, situational, and resource-related supports, including healthy status and financial burden.^30^^,^^32^^,^^34^^,^^36^^,^^39^^,^^41^ Additionally, preferences for disease- and treatment-related information, access to multiple information sources, opportunities for learning through personal or vicarious experiences,^25^^,^^33^^,^^42^ and supportive social discussion with family members, friends, or community groups,^33^^,^^34^^,^^42^ as well as awareness gained through prior personal experience with LST or functional decline,^24^^,^^28^^,^^34^ appeared to support patient engagement in SDM. Personal values and preferences regarding death or care, such as favoring dignity in death and incorporation of individual preferences, were also identified as facilitators.^28–30^

Across the included studies, several patient-related characteristics—including complex health and care needs (eg, severe illness, high care burden) and financial burden—were reported as influencing engagement in SDM in different ways depending on the context.

#### Caregiver-related factors

Among caregiver-related factors, barriers to SDM were primarily related to family-led decision-making dynamics, including situations in which family members made decisions on behalf of patients, exerted pressure to decide, or imposed pressure related to filial piety.^23^^,^^25^^,^^29^^,^^31^^,^^34^^,^^40^ Avoidance of conversations about dying and limited family support further constrained SDM.^27–29^ In a few studies, caregiver characteristics, such as lack of preparedness for bereavement, limited knowledge about disease management, and poorer physical or psychological health, were reported as barriers.^26^^,^^39^

Caregiver-related facilitators included shared involvement of patients and family members in decision-making and respect for patients’ decisions.^23^^,^^33^^,^^37^ In addition, detailed communication with clinicians and a better understanding of the clinical situation, through awareness of treatment ineffectiveness and caregivers’ higher educational level, were also identified as factors that could support caregiver participation in SDM.^29^^,^^30^^,^^41^

#### Clinician-related factors

Among clinician-related factors, major barriers to SDM included a lack of communication skills, discomfort in managing uncertainty, and unclear explanations.^23^^,^^25^^,^^27^^,^^34^^,^^40^ Clinician-centered decision-making practices, including pressure to make decisions and authoritative behaviors,^26^^,^^27^^,^^33^^,^^40^ as well as limited interaction and emotional support,^23^^,^^25^^,^^27^ were also identified. Additionally, one study reported that EOL decisions made close to death—indicated by first DNR orders issued within 29 days before death—limited opportunities for SDM.^31^

Clinician-related facilitators included patient-centered communication practices and respect for patient preferences.^29^^,^^33^^,^^36^ Continuity of care, including involvement of family medicine practitioners or primary care providers, was also reported as supportive of SDM among older adults.^31^^,^^34^

#### Health system–related factors

Among health system-related factors, barriers to SDM were frequently related to a lack of decision-support infrastructure, such as decision-support systems, training or consultation opportunities, or psychological support.^23^^,^^26–29^^,^^40^ Additional barriers reflected resource and structural constraints, including inconsistency in resources across institutions, limited financial support or insurance coverage,^23^^,^^25^^,^^34^ and fragmentation and lack of care across settings and specialities.^25–27^ In several studies, difficulty identifying appropriate disease-related information, the complexity of decision-making processes,^25^^,^^34^ and specific care-setting constraints, such as last admissions to an intensive care unit or high annual treatment volume,^31^^,^^38^ were also reported to impede SDM.

Health system-related facilitators included the availability of hospital palliative care and the involvement of social workers,^31^^,^^32^ as well as the integration of community leadership and local activities.^34^ High-capacity facilities,^38^ consistent, high-quality care, and comfortable care environments^29^ were also identified as supportive.

#### Results summary

**Figure 2:**
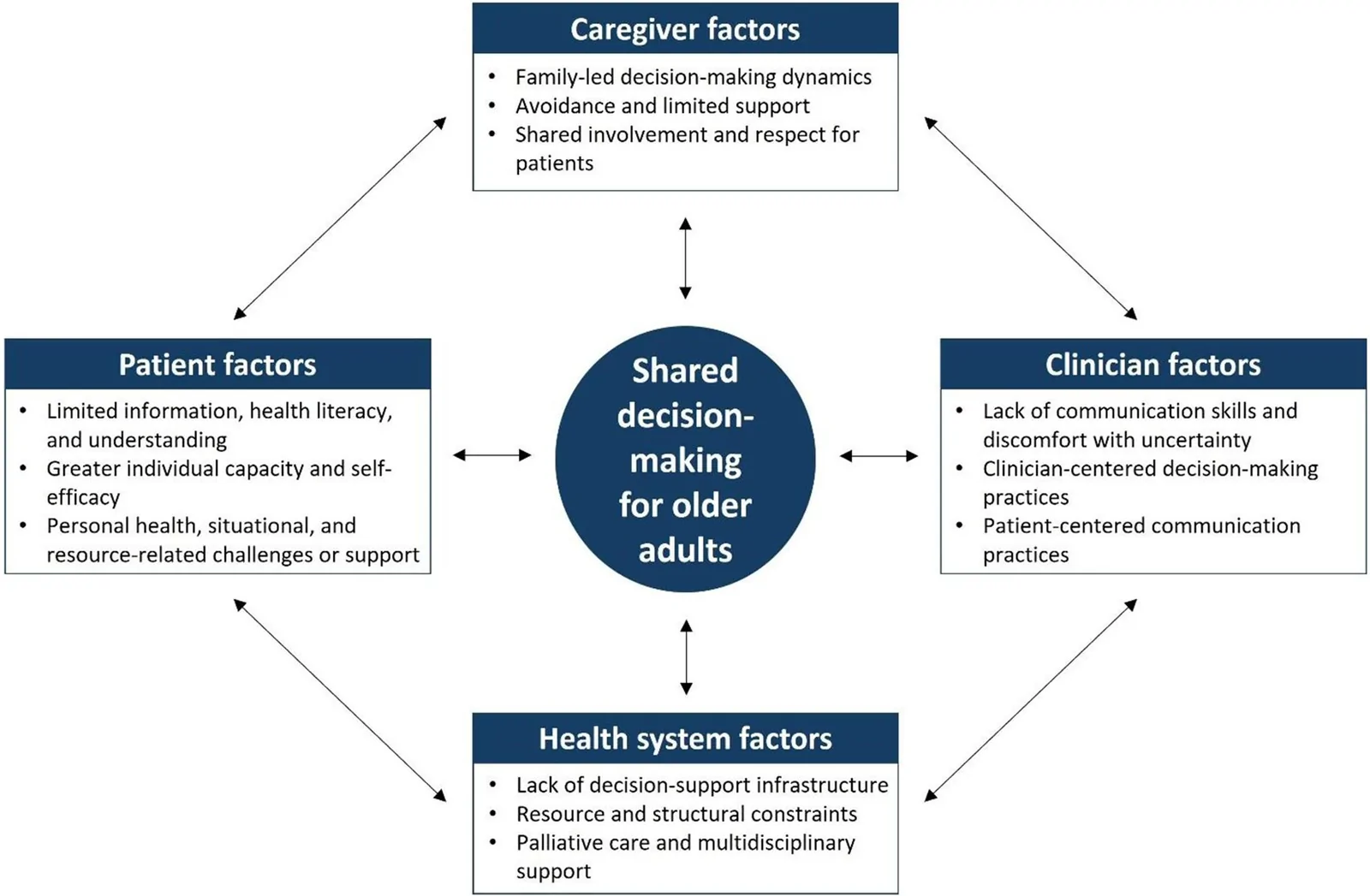
Stakeholder-related factors influencing shared decision-making among older adults in East Asian healthcare settings.

Overall, the findings indicate that barriers and facilitators to SDM among older adults in East Asian healthcare settings arise from multiple, interrelated influences. Factors related to patients, caregivers, clinicians, and the health system collectively shaped opportunities for discussing options, deliberating on treatment decisions, and incorporating patients’ values and preferences into clinical decisions, rather than functioning in isolation. Figure 2 summarizes these interacting factors and illustrates their dynamic relationships across stakeholder groups. Table 3 presents the detailed subcategories of barriers and facilitators identified in this review.

## Discussion and implications

This scoping review has identified multiple barriers and facilitators across patients, caregivers, clinicians, and health systems that affect SDM for older adults in East Asian healthcare settings. Overall, the findings suggest that SDM in this context is influenced by interrelated factors involving multiple stakeholder groups, rather than solely by the communication between individual patients and clinicians. While several barriers and facilitators resemble those identified in Western healthcare systems, the review highlights the prominent roles of family involvement, clinician authority, and constraints within health systems, such as limited supportive infrastructure and resources, and also reveals that existing evidence is heavily concentrated in EOL, LST, and ACP contexts. Taken together, these findings suggest that while many barriers and facilitators to SDM are broadly shared, their expression and relevance in East Asian healthcare settings are influenced by distinct sociocultural norms, relational dynamics, and system-level constraints.

Several barriers and facilitators identified in this review are consistent with findings from prior systematic reviews conducted predominantly in Western contexts. For example, patient-related barriers, such as declining health status, cognitive or physical impairment, psychological distress, and limited health literacy, closely mirror those reported in a prior systematic review.^12^ Similarly, challenges related to clinicians, including time constraints, communication difficulties, and discomfort with uncertainty, as well as health system-related barriers such as limited resources and insufficient organizational support, were evident across both Western and East Asian settings.^10^^,^^12^ These overlaps suggest that many foundational challenges to SDM among older adults are shared across healthcare systems, regardless of geographic or cultural context.

However, despite these shared challenges, the current review also highlights important contextual differences in how SDM is enacted in East Asian healthcare settings. In contrast to much of the Western SDM literature, which typically conceptualizes decision-making as a dyadic interaction between patients and clinicians, with caregiver involvement regarded as optional or supplementary,^2^^,^^5^ the studies included in this review suggest that family members often assume a central role in decision-making, sometimes actively influencing or even replacing patient participation in clinical decision-making.^23^^,^^33^^,^^40^ Rather than reflecting a simple transfer of decision-making authority, this pattern appears to reflect culturally embedded practices in East Asian societies, where collectivist values and family-centered norms position the family as a key factor in healthcare decisions.^7^^,^^10^ For example, qualitative research among Chinese patients undergoing prostate cancer surgery found that treatment decisions were frequently made by family members, occasionally with limited direct patient involvement.^23^ Similarly, a recent scoping review of SDM research in the Korean healthcare system reported that strong family involvement and hierarchical patient–clinician relationships were recurring contextual features influencing SDM implementation.^10^ These findings suggest that family participation is not merely a supplementary element of SDM but represents an important component through which treatment decisions are negotiated in many East Asian healthcare encounters.

The prominence of family involvement observed in this review is consistent with scholarship on Confucian-influenced norms, such as filial piety and family harmony, which emphasize collective responsibility and relational decision-making.^7^^,^^43^ In practice, these norms may encourage families to assume protective roles in medical decision-making, for example, by shaping information disclosure or prioritizing perceived family interests over individual autonomy.^9^ Across the studies included in this review, family involvement emerged as both a barrier and a facilitator to SDM. In some cases, it appeared to facilitate SDM by supporting patients in articulating their values and preferences^33^^,^^37^; in others, it constrained participation by limiting patients’ opportunities to engage meaningfully in decisions.^25^^,^^29^ These findings suggest that family involvement is neither inherently beneficial nor detrimental to SDM. Rather, its influence appears to depend on how family roles are negotiated during clinical encounters and how clinicians and healthcare systems support these interactions.

Importantly, the dual role of family involvement is not unique to East Asian healthcare systems. A prior systematic review in the United States among racial and ethnic minority patients similarly found that SDM may be shaped by cultural and relational dynamics that can either facilitate or hinder engagement, particularly in the context of serious illness.^44^ These parallels caution against simplistic East–West dichotomies and underscore the importance of understanding family involvement as a contextual component of decision-making, rather than a categorical deviation from ideal models of SDM.^7^

In addition to family involvement, our findings also indicate that clinician authority and hierarchical patient–clinician relationships may influence SDM in East Asian healthcare settings. Several studies described clinician-centered decision-making practices, including authoritative behaviors, clinician-driven recommendations, and limited opportunities for interactive communication, which may constrain patient participation.^26^^,^^27^^,^^33^^,^^40^ These dynamics may limit patients’ opportunities to express preferences and engage actively in decision-making, while also affecting how information is communicated and how decision-making roles are negotiated during clinical encounters.^8^^,^^9^ In some East Asian healthcare settings, hierarchical clinical relationships may therefore be closely associated with how SDM is practiced.

An additional distinction concerns the clinical context in which SDM has been studied. Unlike Western-focused systematic reviews, which synthesize SDM across a broad range of decision types and healthcare settings,^12^^,^^45^ the literature included in this review indicates that SDM and SDM-related concepts in East Asian healthcare systems have been predominantly investigated within the domains of EOL care, LST decisions, and ACP. Notably, several studies applied SDM principles primarily within the context of EOL-related planning and treatment decisions, rather than explicitly framing SDM as a distinct decision-making model. This pattern is consistent with findings from a recent scoping review of SDM research in the Korean healthcare system, which identified EOL care as the most prevalent clinical context examined.^10^ While EOL settings may render questions about participation, value prioritization, and decision-making authority particularly visible, this concentration also highlights a substantive limitation of the current East Asian SDM literature. The predominance of EOL-focused research may limit the conceptualization of SDM to critical or terminal decisions and constrain the transferability of existing findings to routine outpatient care, chronic disease management, and other non–EOL clinical contexts. In addition, our findings suggest that relatively limited attention has been given to acute or hyperacute clinical scenarios, such as surgical decision-making, which further narrows the scope of current SDM research in East Asian settings. Addressing this imbalance represents an important direction for future SDM research in East Asian healthcare systems.

### Implications for future practice, policy, and research

Our findings raise important implications for existing SDM frameworks and implementation strategies. Many widely used SDM models, developed primarily within Western healthcare systems, assume a patient–clinician dyadic focus.^5^^,^^46^^,^^47^ While such models provide valuable guidance, the present review suggests that they may be insufficient when applied uncritically to contexts where family involvement and health system constraints play a central role. A broader perspective that incorporates caregiver dynamics and organizational influences may therefore be better suited to capturing the realities of decision-making for older adults in East Asian healthcare systems. Culturally responsive models should treat family participation as a negotiable and potentially supportive component of the decision-making process,^18^^,^^48^ while safeguarding the older person’s preferences and voice.^49^

From an implementation perspective, the implications of these findings extend beyond individual clinician–patient interactions and suggest the need for coordinated strategies across multiple stakeholders to promote SDM among older adults in East Asia. Interventions focused solely on enhancing patient knowledge or clinician communication skills may have limited impact without parallel efforts to engage family caregivers and address organizational barriers, such as limited consultation time and insufficient decision-support infrastructure. Prior implementation research has described the integration of patient decision aids into routine clinical workflows to facilitate structured communication during consultations.^46^ Large-scale implementation initiatives, such as the Making Good Decisions in Collaboration (MAGIC) program, have likewise emphasized multilevel approaches targeting clinicians, patients, and healthcare organizations simultaneously to support sustained adoption of SDM.^50^ For example, the MAGIC program incorporated clinician communication training, patient activation strategies, executive-level organizational support, and adaptation of clinical pathways to embed SDM into routine care. It also emphasized that decision-making is distributed across healthcare teams and family networks rather than confined to a single clinician–patient encounter. These examples illustrate that effective SDM implementation may require not only clinician training, but also organizational support for caregiver engagement and the routine use of decision-support tools within clinical practice.

### Strengths and limitations

This review has several limitations. First, only studies published in English were included, and region-specific databases were not searched. As a result, locally published studies may have been excluded, potentially introducing language and publication bias and limiting the comprehensiveness of the evidence base. This restriction may also have contributed to a relative overrepresentation of studies conducted in specific clinical contexts, such as EOL or LST decision-making. Furthermore, although this review focuses on East Asia as a broad regional category, the findings should be interpreted with caution because healthcare systems and sociocultural contexts vary across countries and regions. In addition, the included studies were conducted in a limited number of countries and regions within East Asia, which may not fully capture the diversity of the region. Additionally, the studies reviewed varied in how SDM was conceptualized, with some focusing explicitly on SDM and others examining related practices such as ACP or EOL decision-making. Although this conceptual heterogeneity is consistent with the exploratory scope of a scoping review, it may have limited comparability across studies and complicated the interpretation of barriers and facilitators attributed specifically to SDM. Lastly, most included studies employed cross-sectional designs, which may limit the interpretation of temporal or causal relationships. Nevertheless, by synthesizing evidence across multiple stakeholder groups, this review provides a comprehensive and context-sensitive overview of barriers and facilitators to SDM among older adults in East Asian healthcare systems.

## Conclusion

This scoping review synthesizes evidence on barriers and facilitators to SDM among older adults in the East Asian healthcare systems and shows that SDM is shaped by interacting influences involving patients, caregivers, clinicians, and health systems. While several of these barriers and facilitators are consistent with those identified in Western contexts, the findings also highlight the important role of family involvement, clinician authority, and structural constraints in SDM for older adults in East Asia. The existing literature has focused largely on EOL care, LST decisions, and ACP, thereby narrowing current understandings of how SDM functions across the wider range of healthcare decisions encountered by older adults. Future research should therefore extend SDM inquiry beyond EOL contexts and explore how culturally responsive approaches that engage patients, families, clinicians, and health systems can support meaningful participation in everyday clinical decision-making across diverse care settings in East Asia.

## Supplementary Material

igag080_Supplementary_Data

## Data Availability

The data for this review were derived from published articles retrieved from 6 databases: CABI Global Health, CINAHL, Embase, PubMed, Scopus, and Web of Science. The search strategies, bibliographic details, and related information are described in this article. The protocol for this review was registered with Open Science Framework (https://osf.io/tx29b/overview).
