# Supplementary material for: Exploring barriers and facilitators to shared decision-making for older adults in East Asian health systems: a scoping review
Source: Innov Aging. 2026 Jul 10;10(9):igag080. doi: 10.1093/geroni/igag080 (PMC13499687; doi:10.1093/geroni/igag080)
Supplement: Supplementary file 1 [file igag080_supplementary_data.pdf]

***Innovation in Aging* Supplementary Material: Lee, Hwang, & Jiang. Exploring barriers and facilitators to shared decision-making for older adults in East Asian Health systems: A scoping review.**

Supplementary Methods. Search Strategies

Concepts/Keywords:
1. Shared decision-making
2. East Asian: China, Japan, Korea, Taiwan, Hong Kong, Macau, and Mongolia
3. Adults ≥ 65

CINAHL: 1 AND 2 AND 3

1. MH "Decision Making, Shared" OR "shared decision making" OR ((MH "Patient Participation" OR MH "Patient Preference" OR "patient participation" OR (patient N2 involvement) OR (patient N2 involved)) AND (MH "Decision Making, Patient" OR MH "Decision Making" OR decision*))
2. MH "East Asia+" OR "east asia*" OR "eastern asia*" OR "hong kong" OR beijing OR china OR chinese OR japan* OR korea* OR macau OR mongolia* OR taiwan* OR tibet OR tokyo
3. MH "Health Services for Older Persons" OR MH "Hospitalization of Older Persons" OR MH "Aged+" OR MH "Geriatrics+" OR "older adult*" OR ageing OR aging OR elder* OR geriatric* OR gerontology OR senior*

Embase: 1 AND 2 AND 3

1. 'shared decision making'/exp OR 'shared decision making':ti,ab,kw OR (('patient participation'/exp OR 'patient preference'/exp OR 'patient participation':ti,ab,kw OR ('patient' NEAR/3 'involvement'):ti,ab,kw OR ('patient' NEAR/3 'involved'):ti,ab,kw) AND ('decision making'/exp OR 'decision*':ti,ab,kw))
2. 'Far East'/exp OR 'east asia*':ti,ab,kw OR 'eastern asia*':ti,ab,kw OR 'korea*':ti,ab,kw OR 'japan*':ti,ab,kw OR 'china':ti,ab,kw OR 'chinese':ti,ab,kw OR 'taiwan*':ti,ab,kw OR 'hong kong':ti,ab,kw OR 'beijing':ti,ab,kw OR 'macau':ti,ab,kw OR 'tibet':ti,ab,kw OR 'tokyo':ti,ab,kw OR 'mongolia*':ti,ab,kw
3. 'aging'/exp OR 'geriatrics'/exp OR 'aged'/exp OR 'elderly care'/exp OR 'longevity'/exp OR 'ageing':ti,ab,kw OR 'aging':ti,ab,kw OR 'older adult*':ti,ab,kw OR 'elder*':ti,ab,kw OR 'senior*':ti,ab,kw OR 'geriatric*':ti,ab,kw OR 'gerontology':ti,ab,kw

Global Health (EBSCO)

("shared decision making" OR ((patient N2 (participation OR involvement OR involved)) AND decision*)) AND ("east asia*" OR "eastern asia*" OR "hong kong" OR beijing OR china OR chinese OR japan* OR korea* OR macau OR mongolia* OR taiwan* OR tibet OR tokyo) AND

("older adult*" OR ageing OR aging OR elder* OR geriatric* OR gerontology OR senior*)

PubMed: 1 AND 2 AND 3

1. "Decision Making, Shared"[Mesh] OR "shared decision making"[tiab] OR (("Patient Participation"[Mesh] OR "Patient Preference"[Mesh] OR "Patient Participation"[tiab] OR "Patient involvement"[tiab:~2] OR "Patient involved"[tiab:~2]) AND ("Decision Making"[Mesh] OR decision*[tiab]))
2. "Asia, Eastern"[Mesh] OR "east asia*"[tiab] OR "eastern asia*"[tiab] OR Korea*[tiab] OR Japan*[tiab] OR China[tiab] OR Chinese[tiab] OR Taiwan*[tiab] OR "Hong Kong"[tiab] OR Beijing[tiab] OR Macau[tiab] OR Tibet[tiab] OR Tokyo[tiab] OR Mongolia*[tiab]
3. "Aging"[Mesh] OR "Geriatrics"[Mesh] OR "Aged"[Mesh] OR "Health Services for the Aged"[Mesh] OR "Longevity"[Mesh] OR ageing[tiab] OR aging[tiab] OR "older adult*"[tiab] OR elder*[tiab] OR senior*[tiab] OR geriatric*[tiab] OR gerontology[tiab]

Scopus

("shared decision making" OR ((patient W/2 (participation OR involvement OR involved)) AND decision*)) AND ("east asia*" OR "eastern asia*" OR "hong kong" OR beijing OR china OR chinese OR japan* OR korea* OR macau OR mongolia* OR taiwan* OR tibet OR tokyo) AND ("older adult*" OR ageing OR aging OR elder* OR geriatric* OR gerontology OR senior*)

Web of Science

TS=(("shared decision making" OR ((patient NEAR/2 (participation OR involvement OR involved)) AND decision*)) AND ("east asia*" OR "eastern asia*" OR "hong kong" OR beijing OR china OR chinese OR japan* OR korea* OR macau OR mongolia* OR taiwan* OR tibet OR tokyo) AND ("older adult*" OR ageing OR aging OR elder* OR geriatric* OR gerontology OR senior*))

Supplementary Table 1. Detailed Study Characteristics

First author (year)	Study aim	Study design/methods	Setting (country or region)	Sample size/Age (years)
Cao (2022) (23)	To investigate the factors that impact the degree of participation in surgical decision-making among Chinese prostate cancer patients	Qualitative study (phenomenological approach)<br><br>Semi-structured interviews<br>Data collection: Sep 2018 to Aug 2019<br>Colaizzi's seven-step method for analysis	1 general tertiary hospital (China)	Total *n*=50<br>- 34 patients with prostate cancer<br>- 16 medical and nursing staff<br><br>Mean age: 65.3
Chen (2022) (31)	To understand the fact of terminal cancer patients' autonomous do-not-resuscitate (DNR) decision-making	Secondary analysis of longitudinal data<br><br>Data collection: Jan 2017 to Dec 2018<br>Multivariate logistic regression	1 tertiary public medical center's medication system (Taiwan)	Total *n*=1338<br>- patients with cancer who died in a hospital<br><br>Mean age: 66.8
Chong (2024) (40)	To understand decision-making experiences regarding kidney transplantation (KT) among older adults with end-stage renal disease (ESRD)	Qualitative descriptive study<br><br>Semi-structured interviews<br>Data collection: Mar to Apr 2021<br>Thematic analysis	1 university hospital (South Korea)	Total *n*=12<br>- patients underwent KT<br><br>Mean age: 67.4
Chu (2018) (32)	To identify factors associated with advance directives (AD) completion during advance care planning among hospitalized patients with chronic life-limiting illness	Secondary data analysis<br><br>Data collection: Apr 2015 to Jan 2016<br>Multivariable logistic regression	1 public hospital (Taiwan)	Total *n*=1411<br>- patients with chronic life-limiting illness or functional impairment limiting their ability to complete activities of daily living<br><br>Mean age: 78.2
Chu (2011) (42)	To describe knowledge and preferences of older adults regarding advance directives (AD) and end-of-life (EOL) care decisions, and to investigate the predictors of preference for AD and end-of-life care in nursing homes	Cross-sectional survey<br><br>Data collection: Jul 2007 to Jul 2008<br>Multivariate logistic regression	140 nursing homes (Hong Kong, China)	Total *n*=1600<br>- older adults (≥65 years) residing in nursing homes<br><br>Mean age: 82.4
Chuang (2024) (33)	To explore the shared decision-making related experiences and perspectives of patients with degenerative joint disease (DJD)	Qualitative study<br><br>Semi-structured interviews<br>Data collection: Oct 2021 to May 2022<br>Thematic analysis	1 regional teaching hospital (Taiwan)	Total *n*=21<br>- outpatients with at least three visits for DJD<br><br>Mean age: 69.9
Fang (2025) (24)	To investigate the impact of life-sustaining treatment (LST) experiences (cardiopulmonary resuscitation, tracheal intubation, tube feeding, expensive antibiotics use, or hemodialysis) on EOL decision-making among older adults in nursing homes	Cross-sectional survey<br><br>Data collection: Aug 2023<br>Logistic regression, marginal effects analysis	1 public nursing home (China)	Total *n*=571<br>- older adults with disability, dementia, or those over 80 years<br><br>Mean age: 86.9

First author (year)	Study aim	Study design/methods	Setting (country or region)	Sample size/Age (years)
Guo (2024) (25)	To explore the experiences and needs of older adults with stroke during rehabilitation decision-making	Qualitative study (phenomenological approach)<br><br>Semi-structured interviews<br>Data collection: Jul 2023 to Nov 2023<br>Inductive thematic analysis	1 tertiary care hospital (China)	Total *n*=31<br>- older adults (≥60 years) with stroke<br><br>Mean age: Not reported (but more than 50% of participants were older than 70 years)
Hsu (2025) (34)	To investigate the facilitators and barriers to participating in advance care planning during the COVID-19 pandemic among community-dwelling older adults with chronic diseases	Qualitative study<br><br>Semi-structured interviews<br>Data collection: Nov 2021 to Sep 2022<br>Thematic analysis	1 community center (Taiwan)	Total *n*=36<br>- 13 community-dwelling older adults (≥65 years) with at least one chronic disease<br>- 12 primary family caregivers<br>- 11 community nurses<br><br>Mean age: 71.7
Kanakubo (2024) (36)	To examine variation in patient-centered care (PCC) across dialysis facilities and the association between PCC and ACP preparedness among patients on hemodialysis	Multicenter cross-sectional survey<br><br>Data collection: Apr 2022 to Feb 2023<br>Modified Poisson regression analysis	6 medical institutions (Japan)	Total *n*=453<br>- patients undergoing maintenance hemodialysis at the participating facilities<br><br>Mean age: 69
Komatsu (2022) (37)	To examine the association of recipient- and caregiver-related factors with decision-making involvement of community-dwelling older care recipients	Secondary data analysis<br><br>Data collection:<br>2003<br>Multivariable logistic regression	2 cities (Japan)	Total *n*=1286<br>- 643 older adults (≥65 years) covered by the long-term care insurance system<br>- 643 primary caregivers<br><br>Mean age: Not reported
Lin (2025) (35)	To investigate the association between<br>medical decision-making participation self-efficacy and ACP engagement among older adults receiving home-based medical care	Cross-sectional secondary analysis<br><br>Data collection: Nov 2019 to Dec 2022<br>Multivariable logistic regression	Communities (Taiwan)	Total *n*=408<br>- older adults (≥50 years) receiving home-based medical care for more than two months<br><br>Mean age: 80.4
Liu (2025) (26)	To explore willingness, needs, and factors influencing participation in medication decision-making among older adults with multimorbidities, caregivers, and healthcare professionals	Qualitative study (Phenomenological approach)<br><br>Semi-structured interviews<br>Data collection: Mar 2024 to Jul 2024<br>Colaizzi 7-step analysis	1 tertiary hospital (China)	Total *n*=28<br>- 12 older adults with multimorbidities<br>- 9 caregivers<br>- 7 healthcare professionals<br><br>Mean age: 65
Shibagaki (2025) (38)	To examine SDM perceptions, preferences, and factors influencing SDM perception among patients with chronic kidney disease (CKD)	Cross-sectional survey<br><br>Data collection: Oct 2022 to Sep 2024<br>Poisson regression	49 medical facilities (Japan)	Total *n*=475<br>- adult patients with stage 5 CKD, who had selected one of the modalities of renal replacement therapy<br><br>Mean age: 67.4
Shin (2018) (41)	To explore factors associated with preferences for patients'	Cross-sectional survey	11 cancer centers (South Korea)	Total *n*=716<br>- 358 older adults (≥60 years)

First author (year)	Study aim	Study design/methods	Setting (country or region)	Sample size/Age (years)
	active participation in treatment decision-making and the degree to which patients and family members agree on preferences	Data collection: 2014<br>Multivariable logistic regression		with gastric, lung, or colorectal cancer<br>- 358 adult family caregivers<br><br>Mean age: 71.1
Shinada (2022) (39)	To examine the frequency and circumstances of caregiver difficulties in decision-making during EOL care for patients with cardiovascular diseases, their determinants, and associations with psychological distress in the bereaved caregivers	Cross-sectional survey<br><br>Data collection: Jan 2014 to Dec 2016<br>Bivariate analysis	10 tertiary care centers (Japan)	Total *n*=266<br>- bereaved caregivers of older patients with cardiovascular diseases<br><br>Median age (patients): 82 (73-88)
Wang (2025) (27)	To explore the perception of SDH during cardiac rehabilitation among coronary artery bypass grafting (CABG) postoperative patients	Qualitative study (Phenomenological approach)<br><br>Semi-structured interviews<br>Data collection: Feb 2025 to Apr 2025<br>Colaizzi 7-step analysis	3 tertiary hospitals (China)	Total *n*=19<br>- patients who have undergone CABG<br><br>Mean age: 72.2
Yang (2021) (28)	To investigate the acceptance and influencing factors of ACP for community-dwelling older adults with chronic diseases	Mixed-method study (Convergent parallel design)<br><br>Data collection: Sep 2019 to Jan 2020<br>Interviews: thematic analysis<br>Survey: multiple linear stepwise regression analysis	3 communities (China)	Total *n*=471 (Survey)<br>- older adults (≥60 years) with chronic diseases who had made at least two visits to community health service centers<br>- Among survey participants, 14 conducted interviews<br><br>Mean age: Not reported (but more than 50% of participants were older than 70 years)
Ye (2025) (29)	To explore hospice and palliative care nursing (HPCN) preferences among older residents in nursing homes	Qualitative study (Grounded theory approach)<br><br>Semi-structured interviews<br>Data collection:<br>Oct 2021 to Jan 2022<br>Corbin and Strauss's method	5 nursing homes (China)	Total *n*=20<br>- older adults with at least one chronic disease or cancer, ensuring progressive conditions requiring HPCN engagement<br><br>Mean age: 77.8
Zhu (2023) (30)	To explore and compare preferences and attitudes regarding LST among community-dwelling older adults with chronic conditions and their family caregivers	Cross-sectional survey<br><br>Data collection: Jan 2019 to Nov 2019<br>Bivariate analysis	4 communities (China)	Total *n*=300<br>- 150 older adults (≥60 years) with a chronic condition<br>- 150 family caregivers<br><br>Mean age: 71

Supplementary Table 2. Barriers and facilitators to shared decision-making at multiple stakeholder groups

**Stakeholder groups**	**Barriers**	**Facilitators**
Patient	1) Limited information, health literacy, and understanding (*n*=7)<br>・Limited knowledge or information (23,25-27,29,40)<br>・Low health literacy or educational level (23,25,31)<br>・Aging-related cognitive or physiological impairment (23,27)<br>2) Personal health, situational, and resource-related challenges (*n*=8)<br>・Complex health and care needs (e.g., severe illness, high care burden) (31,37,39,40,42)<br>・Financial burden (26,38)<br>・Lack of psychosocial support (29)<br>3) Psychological and emotional distress (*n*=5)<br>・Poor psychological health (e.g., worry, regret, anxiety, depression) (23,25,27,40)<br>・Fear of medical authority or death (27,28)<br>4) Constrained autonomy and interactions with clinicians (*n*=5)<br>・Dependence on suggestions from clinicians (25-27,33,40)<br>・Lack of personalized guidance (27)<br>・Indirect rejection or postponement of clinician-prescribed treatment plans (33)<br>5) Avoidance or reluctance toward EOL discussion (*n*=3)<br>・Avoidance of EOL conversation (29,34)<br>・Religious reluctance (34)<br>・Passive attitude toward death (40)	1) Greater individual capacity and self-efficacy (*n*=6)<br>・High educational level (24,28,41)<br>・High self-care ability (26,28,42)<br>・High self-efficacy in medical decision-making (35)<br>2) Personal health, situational, and resource-related support (*n*=6)<br>・Complex health and care needs (e.g., severe illness, high care burden) (30,32,36,39,41)<br>・Healthy status (30)<br>・Financial burden (34)<br>3) Information preferences, access, and learning opportunities (*n*=3)<br>・Preferences for information about disease and treatment (33,42)<br>・Access to multiple information sources (25)<br>・Personal or vicarious learning experiences (33)<br>4) Supportive social discussion (*n*=3)<br>・Discussion with family, friends, or community groups (33,34,42)<br>5) Prior experience-based awareness (*n*=3)<br>・Witnessed experiences of life-sustaining treatment (24,28,34)<br>・Early recognition of functional decline (34)<br>6) Personal values favoring dignity in care (*n*=3)<br>・Preference for dignity in death (28-30)<br>・Incorporation of personal preferences (29)
Caregiver	1) Family-led decision-making dynamics (*n*=6)<br>・Decision-making on behalf of patients (23,25,29,34,40)<br>・Family pressure to decide (40)<br>・Pressure related to filial piety (34)<br>・Multiple children (31)<br>2) Avoidance and limited support (*n*=3)<br>・Avoidance of EOL conversation (28)<br>・Limited family support (27,29)<br>3) Lack of caregiver preparedness, knowledge, and health (*n*=2)<br>・Unpreparedness for bereavement (39)	1) Shared involvement and respect for patients (*n*=3)<br>・Joint decision-making by patients and family (23,33)<br>・Respect for patient decisions (37)<br>2) Detailed communication and understanding of the clinical situation (*n*=3)<br>・Detailed communication with clinicians (29)<br>・Awareness of treatment ineffectiveness (30)<br>・High educational level (41)

Stakeholder groups	Barriers	Facilitators
	・Lack of knowledge about disease management (26)<br>・Poor health status (39)	
Clinician	1) Lack of communication skills and discomfort with uncertainty (*n*=5)<br>・Lack of communication skills (23,34)<br>・Discomfort in discussing uncertainty (23)<br>・Unclear explanations from clinician (25,27,40)<br>2) Clinician-centered decision-making practices (*n*=4)<br>・Clinician pressure to decide (40)<br>・Authoritative behaviors (33)<br>・Clinician-centered decision-making (26,27)<br>3) Limited interaction and emotional support (*n*=3)<br>・Limited consultation time or time shortage (23,27)<br>・Limited interactive communication with patients (25,27)<br>・Lack of emotional support for patients (27)<br>4) Delayed timing of EOL decisions (*n*=1)<br>・Late initiation of EOL discussions (first DNR order 29 days before death) (31)	1) Patient-centered communication practices (*n*=3)<br>・High-quality patient-centered care (e.g., discussion of multiple options, personalized information) (29,33,36)<br>・Respect for patient preferences (33)<br>2) Continuity of care (*n*=2)<br>・Involvement of family medicine practitioners on the last admission service (31)<br>・Involvement of primary care providers in the community (34)
Health system	1) Lack of decision-support infrastructure (*n*=6)<br>・Lack of decision-support system or training (23,26-29,40)<br>・Challenges in providing comprehensive treatment (26)<br>・Lack of consultation opportunities or psychological support (27)<br>2) Resource and structural constraints (*n*=3)<br>・Resource inconsistency across institutions (23)<br>・Limited financial support and insurance coverage (25,34)<br>3) Fragmentation and lack of care integration (*n*=3)<br>・Lack of system integration across care settings (25,27)<br>・Lack of connection among healthcare professionals across specialties (26)<br>4) Information barriers and complexity (*n*=2)<br>・Difficulties in identifying appropriate disease-related information (25)<br>・Complexity of the decision-making process (25,34)<br>5) Care-setting constraints (*n*=2)<br>・Last admission to an intensive care unit (31)<br>・High annual treatment volume (38)	1) Palliative care and multidisciplinary support (*n*=2)<br>・Availability of hospital palliative care (31)<br>・Involvement of social workers in care planning (32)<br>2) Community integration (*n*=1)<br>・Community leadership and local activity integration (34)<br>3) High facility capacity or volume (*n*=1)<br>・High-capacity facilities (38)<br>4) Quality and stability of care environment (*n*=1)<br>・Consistent, high-quality, and comfortable EOL care (29)

Note. DNR = do-not-resuscitate; EOL = end-of-life.
